# A Novel MZI Fiber Sensor with Enhanced Curvature and Strain Sensitivity Based on Four-Core Fiber

**DOI:** 10.3390/mi15121427

**Published:** 2024-11-27

**Authors:** Xiaojun Zhu, Feijie Chen, Haoran Zhuang, Jiayi Qian, Hai Liu, Juan Cao, Yuechun Shi, Xia Wang, Wuming Wu

**Affiliations:** 1 School of Microelectronics and Integrated Circuits (Jiangsu Key Laboratory of Semi. Dev. & IC Design, Package and Test), Nantong University, Nantong 226019, China; zhuxj0122@ntu.edu.cn; 2 School of Information Science and Technology, Nantong University, Nantong 226019, China; 2330320007@stmail.ntu.edu.cn (F.C.); 2330310026@stmail.ntu.edu.cn (H.Z.); 2230320003@stmail.ntu.edu.cn (J.Q.); cj@ntu.edu.cn (J.C.); 3 School of Information and Control Engineering, China University of Mining and Technology, Xuzhou 221116, China; sieeoe@cumt.edu.cn; 4 Yongjiang Laboratory, Ningbo 315202, China; yuechun-shi@ylab.ac.cn; 5 School of Electronic Science and Engineering, Hunan Institute of Information Technology, Changsha 410151, China

**Keywords:** fiber optic sensor, Mach–Zehnder interferometer, four-core fiber, no-core fiber, curvature sensor

## Abstract

We present a high-sensitivity curvature and strain Mach–Zehnder interferometer (MZI) fiber sensor based on a configuration of no-core fiber (NCF) and four-core fiber (FCF). We used an optical fiber fusion splicer to directly splice a segment of FCF between two segments of NCF, with both the FCF and NCF made of SiO_2_, where the FCF exhibits multi-path interference characteristics that allow for higher sensitivity. The NCF, with its self-focusing property, excites higher-order modes, which split and transmit it into the four cores of the FCF. The experimental results show that within a curvature range of 0.0104 m^−1^–0.1515 m^−1^, the maximum sensitivity can reach −78.04 dB/m^−1^ with a high linear value of ~0.99. Additionally, the strain response is also experimentally studied. In the range of 0–600 με, the maximum strain sensitivity is −6.49 pm/με. The sensor demonstrates high curvature and strain sensitivity, indicating its potential applications in sensing measurements.

## 1. Introduction

Compared to traditional sensors, optical fiber sensors have advantages such as resistance to electromagnetic interference, good flexibility, fast response time, and resistance to chemical corrosion. They have been widely used to measure various physical parameters, such as curvature [1], strain [2], refractive index (RI) [3], temperature [4], and humidity [5]. Among them, the collection of curvature and strain information plays a vital role in environmental monitoring, medical and biochemical engineering, the food industry, and other fields. In the past few years, in-line Mach–Zehnder interferometers (IMZIs) have attracted widespread attention [6,7]. Compared to traditional MZIs, the IMZI centralizes the transmission arm into a single fiber and induces interference by exciting higher-order modes. To enhance the sensitivity of sensors, various efforts have been employed, such as using tapered fibers [8], offset fiber splicing [9], and special optical fibers [10]. Multi-core optical fibers, as a type of specialty fiber, consist of multiple cores arranged together, allowing it to exceed the transmission capacity limitations of single-mode fibers in space division multiplexing optical communication. Additionally, the presence of multiple cores enables the support of more transmission modes, which enhances signal transmission efficiency and sensing performance while reducing the effects of mode interference. As a result, well-designed multi-core fiber sensors can achieve higher sensitivity. In 2018, Tongtong Zhao et al. proposed a new sensor based on dual-core photonic crystal fibers for simultaneously measuring curvature, strain, and temperature. The maximum sensitivities of curvature and strain were 10.89 nm/m^−1^ and 1.24 pm/με, respectively [11]. In 2021, Zhaojun Liu et al. proposed an MZI sensor based on a combination of seven-core optical fiber and fiber Bragg grating, with a maximum strain sensitivity of −2.47 pm/με [12]. In 2022, Yinggang Liu et al. proposed a sensor based on seven-core optical fiber with a curvature sensitivity of 10.22 dB/m^−1^ [13].

However, sensors based on dual-core photonic crystal fiber require higher costs, while FCF is more cost-effective while enabling multi-path transmission. Recently, some FCF-based IMZI sensors have been reported in the field of curvature and strain sensing. For example, in 2017, Chao Li et al. proposed an optical fiber sensor integrating FCF and fiber Bragg grating for the simultaneous measurement of strain, RI, and temperature; the maximum strain sensitivity obtained in the strain range of 0 to 2000 με was −1.75 pm/με [14]. In 2020, Runtao Yang spliced a section of FCF between two coupling sections, where the coupling section was composed of few-mode fiber, multimode fiber, and large core diameter multimode fiber. Its maximum curvature sensitivity was 20.77 nm/m^−1^ in the curvature range of 0.2983 m^−1^ to 0.7916 m^−1^ [15]. In 2022, Xinghu Fu et al. proposed a dual-parameter sensor for temperature and strain based on the combination of tapered FCF and multimode fiber, featuring an SMF-MMF-FCF-SMF structure. A conical shape was formed at the fusion point between the FCF and SMF, creating an optical fiber sensor based on MZI. The results indicated that within the strain range of 0–800 με, the maximum strain sensitivity achieved was −6.76 pm/με [16]. Unfortunately, although FCF improves the curvature and strain sensitivity of these sensors, these sensors have high costs, and the manufacturing process of the coupling section is relatively complex, leading to lower coupling efficiency. Thanks to the self-focusing properties of NCF, the coupling efficiency of light can be effectively improved. In 2022, Jiacheng Sun et al. proposed a multi-parameter measurement sensor based on NCF-SMF-NCF. In their configuration, the two segments of NCF acted as couplers. When light propagates into the NCF, the NCF excites higher-order modes and efficiently couples the light into the SMF [17].

In this paper, we propose an IMZI based on FCF, which can be easily fabricated by splicing a single FCF segment between two NCFs. The two NCFs act as a splitter and a coupler, respectively. The self-focusing characteristics of NCF are utilized to efficiently couple light into the four cores of the FCF, enhancing coupling efficiency, enabling multi-path transmission, and improving sensitivity. In the curvature and strain measurement experiments, a maximum intensity sensitivity of −78.04 dB/m^−1^ was achieved within a curvature range of 0.0104 m^−1^ to 0.1515 m^−1^, and a maximum strain sensitivity of −6.49 pm/με was obtained within a strain range of 0 to 600 με. The multi-path coupling and high-intensity sensitivity of the sensor are realized through the use of NCF, which offers the advantages of an all-fiber structure, ease of fabrication, and compact size. This approach provides a new method for achieving efficient coupling in fiber-based MZI sensors.

## 2. Manufacturing and Sensing Principles

**Figure 1:**
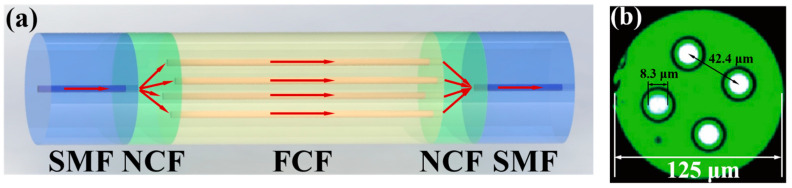
(**a**) Schematic diagram of the proposed sensor; (**b**) cross-sectional image of FCF (provided by EVERFOTON).

The schematic diagram of the proposed sensor structure is shown in Figure 1a. The NCF and FCF are directly fused and sandwiched between two sections of SMF. The core/cladding diameter of the SMF used in the experiment is 8.2/125 μm. The cladding diameter of the NCF (EVERFOTON, Wuhan, China) is 125 μm, and the refractive index is 1.444. Figure 1b shows the cross-section of the FCF. We can see that the FCF (EVERFOTON) contains four identical cores with a square shape distribution, where the distance between two adjacent cores is 42.4 μm. The cores and cladding diameter of the FCF are 8.3 μm and 125 μm, respectively.

When light is incident from SMF to NCF, higher-order modes are excited due to the mismatch between the SMF and NCF core diameters, as shown in Figure 1a. Since NCF has self-focusing properties, light can be coupled into the four cores of FCF. In FCF, the optical path difference is caused by the effective refractive index difference between the core and the cladding, and interference eventually occurs. Compared with the higher-order modes transmitted in the cladding, the core mode transmitted in the FCF core plays a dominant role. Therefore, the FCF sensor can be analyzed as a Mach–Zehnder interference model.

**Figure 2:**
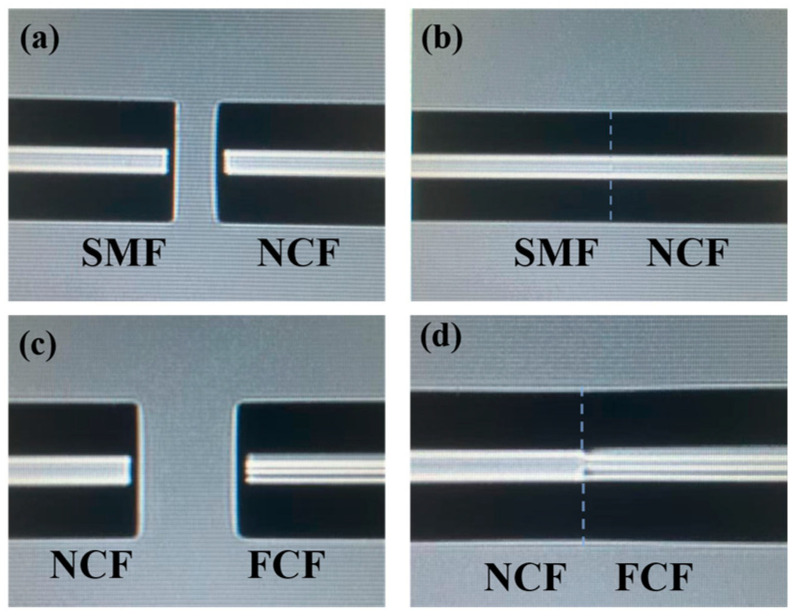
(**a**) SMF and NCF before fusion; (**b**) SMF and NCF after fusion; (**c**) NCF and FCF before fusion; and (**d**) NCF and FCF after fusion.

We use an optical fiber fusion splicer (OFFS, FITEL S178C, Tokyo, Japan) for fusion preparation, and the fusion procedure adopts automatic fusion. All fused fibers should be de-coated and cut flat before fusion. First, the two segments of NCF are fused with the SMF at the input and output ends, as shown in Figure 2a. The SMF and the NCF are placed in the OFFS, and the discharge mode of the OFFS is set to SM-MM, with a discharge intensity of 150. After automatic fusion, the result is shown in Figure 2b. Then, the FCF and NCF are placed in the OFFS, the discharge mode of the OFFS is adjusted to MM-MM, and after automatic fusion, the result is as shown in Figure 2d.

The optical coupling intensity between different cores of the proposed sensor can be express as [18]

(1) $$I=\sum_{n=1}^{4}I_{n}+2\sum_{n=1}^{3}\sum_{m=2,m\neq n}^{4}\sqrt{I_{n}I_{m}}\cdot \cos\left(\emptyset_{n,m}\right),$$

where I_n_ and I_m_ are the intensities of arbitrary two core modes. And $\emptyset_{n,m}$ is the phase difference between them, which can be expressed by

(2) $$\emptyset_{n,m}=2\pi L\cdot \Delta n_{\mathrm{eff}}^{n,m}/\lambda ,$$

where L is the length of the FCF and $\Delta n_{\mathrm{eff}}^{n,m}$ is the difference in effective refractive index (RI) between the two core modes. If the phase difference satisfies $\emptyset_{n,m}=\left(2t+1\right)\pi$, where t = 1, 2, 3, …, the wavelength of the interference valley can be expressed as

(3) $$\lambda_{t}=\frac{2\Delta n_{\mathrm{eff}}^{n,m}L}{2t+1}.$$

When strain is applied along the fiber axis, the length of the MZI will be stretched and the resonant frequency will decrease. The wavelength shift can be expressed as [19]

(4) $$\Delta \lambda_{t}=\left[1+\left(\frac{L}{\Delta n_{\mathrm{eff}}^{n,m}}\right)\left(\frac{\partial \left(\Delta n_{\mathrm{eff}}^{n,m}\right)}{\partial L}\right)\right]\lambda_{t}\varepsilon ,$$

where ε is the strain. It can be seen that the strain sensitivity is closely related to the ${\Delta n}_{\mathrm{eff}}^{n,m}$ caused by the extended FCF length, that is, $\partial \left(\Delta n_{\mathrm{eff}}^{n,m}\right)/\partial L$. At the same time, under the action of strain ε, the proposed sensor will have a slight physical deformation, and the output light intensity will also change slightly.

**Figure 3:**
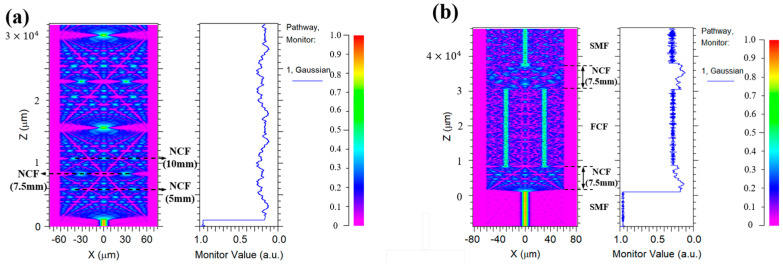
(**a**) Light field distribution of NCF; (**b**) light field distribution of the proposed sensor.

**Figure 4:**
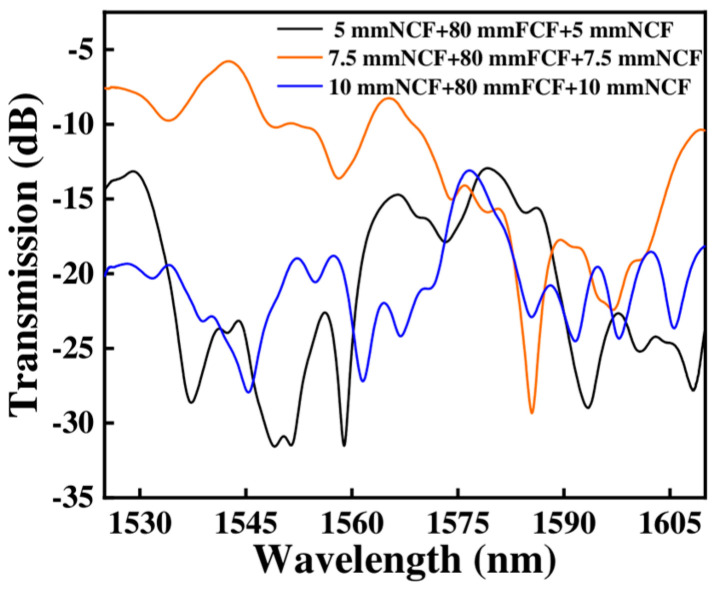
Transmission spectra of sensors with three different NCF lengths.

To better understand the coupling between NCF and FCF, we analyzed the light distribution of the proposed sensor with different lengths. First, we used Rsoft software (Version 2018.12) to simulate light transmission in the NCF, where the refractive index of air and NCF are set to 1.0003 and 1.444, and the transmission wavelength is set at 1550 nm, respectively, as shown in Figure 3a. We can see that the light field distribution of the NCF will exhibit a self-focusing effect when the NCF length is 5 mm, 7.5 mm, and 10 mm, respectively. Meanwhile, the central plot of Figure 3a represents the intensity of light at different positions of the monitor when light transmits through the NCF. Among them, when the NCF length is 7.5 mm, the light will produce two self-focusing points that correspond to the distribution of the FCF, which can be used to couple the light into the multi-core fiber to improve the coupling efficiency. Figure 3b shows the light field distribution of the sensor when the length of the NCF is 7.5 mm, and how the light intensity varies with the monitor. It can be observed that the light intensity in the cores of the FCF is higher, indicating that light can be effectively coupled into the four cores of the FCF due to the self-focusing effect of the NCF. A validation experiment has been employed with different lengths of NCF, as shown in Figure 4. We can see that although all the sensors appear to show the interference phenomenon, the sensor has the lowest loss when the NCF length is 7.5 mm, which means that the light has more success coupling light into the four cores of the FCF and the core of the SMF when the NCF length is 7.5 mm.

## 3. Experiments and Measurement

### 3.1. Curvature Measurement

Three sensors with different FCF lengths (Sensor 1, L = 80 mm; Sensor 2, L = 90 mm; Sensor 3, L = 100 mm) have been conducted to analyze the sensing properties of the proposed sensor.

**Figure 5:**
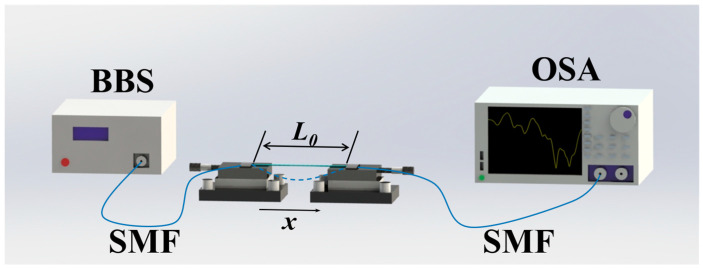
Schematic diagram of curvature experimental device.

The curvature sensing experimental setup is shown in Figure 5. In the curvature measurement experiment, the entire sensor assembly, including NCF, FCF, and NCF, is bent to study the response of the whole sensor structure to curvature. The two ends of the sensor mount on the optical fiber fixtures, where the input port is connected to a broadband light source (BBS, OPEAK LSM-ASE-CF13, Tianjin, China) and the output port connects to a spectrum analyzer with a resolution of 0.02 nm (OSA, Yokogawa AQ6370D, Tokyo, Japan). In the experiment, the curvature can be obtained by adjusting the position of the left fixture, and the curvature can be calculated by

(5) $$C=\frac{1}{R}=\sqrt{\frac{24x}{{\left(L_{0}-x\right)}^{3}}},$$

where L_0_ is the initial distance, R is the radius of curvature, x is the offset, and C is the curvature.

**Figure 6:**
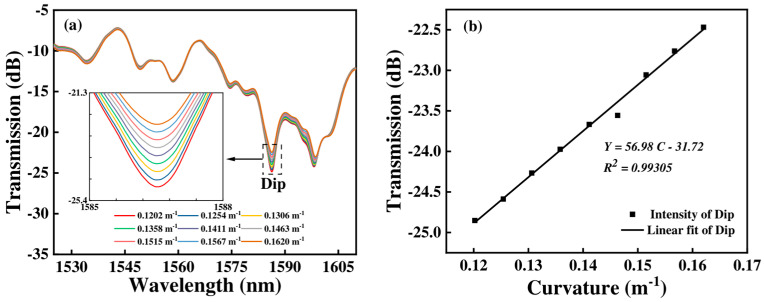
(**a**) The transmission spectra of Sensor 1 with various curvatures; (**b**) the corresponding curvature response of intensity.

When the length of the FCF is 80 mm, the transmission spectra and the linear fitting curve of the proposed sensor are as shown in Figure 6. We can see that when the curvature gradually increases, the wavelength shift in the transmission spectra is narrow and mainly present in the intensity change. When the curvature varies from 0.1202 m^−1^ to 0.1620 m^−1^, the dip intensity changes from −24.852 dB to −22.467 dB. The curvature sensitivity of Sensor 1 in terms of intensity is 56.98 dB/m⁻^1^, as shown in Figure 6b.

**Figure 7:**
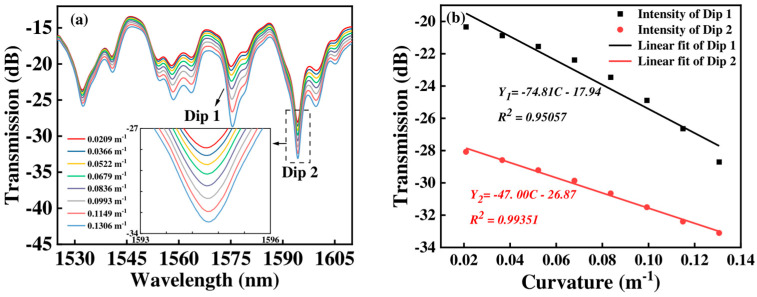
(**a**) The transmission spectra of Sensor 2 with curvatures changing; (**b**) the corresponding curvature responses of intensity.

When the FCF length increases to 90 mm, the transmission spectra and the linear fitting curve of Sensor 2 under different curvature conditions are shown in Figure 7. With the curvature increasing, the intensity of transmission spectra undergoes a large change. When the curvature increases from 0.0209 m^−1^ to 0.1306 m^−1^, the intensity of Dip 1 and Dip 2 change from −20.338 dB to −28.704 dB, and −28.077 dB to −33.104 dB, respectively. The corresponding linear fitting coefficient with intensity is 0.95057 for Dip 1, and 0.99351 for Dip 2, respectively, as shown in Figure 7b. The maximum curvature sensitivity of Sensor 2 is −74.81 dB/m⁻^1^.

**Figure 8:**
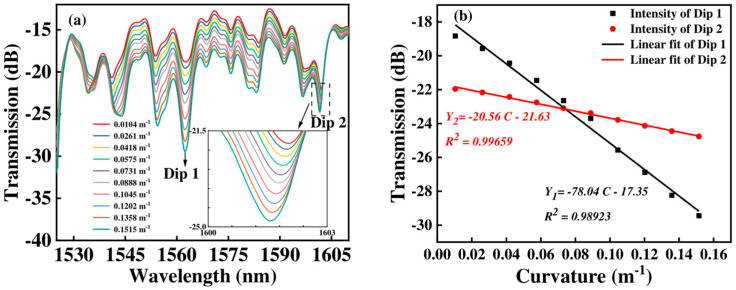
(**a**) The transmission spectra of Sensor 3 with curvatures changing; (**b**) the corresponding curvature responses of intensity.

For Sensor 3, we can see that when the curvature changes from 0.0104 m^−1^ and 0.1515 m^−1^, the intensity of Dip 1 and Dip 2 change from −18.825 dB to 29.444 dB, and −21.956 dB to −24.763 dB, respectively, as shown in Figure 8a. With the curvature changing, the linear fitting coefficient of intensity is 0.98923 for Dip 1 and 0.99659 for Dip 2, respectively, as shown in Figure 8b. From Figure 8, we can obtain that the maximum curvature sensitivity of Sensor 3 is −78.04 dB/m⁻^1^.

By comparing Figure 6 to Figure 8, we can obtain that the maximum curvature sensitivity is −78.04 dB/m^−1^ when the FCF length is 100 mm. Furthermore, the curvature changes primarily affect the intensity rather than the wavelength. That is to say, in the measurement of curvature, the shift in wavelength can be ignored.

### 3.2. Strain Measurement

**Figure 9:**
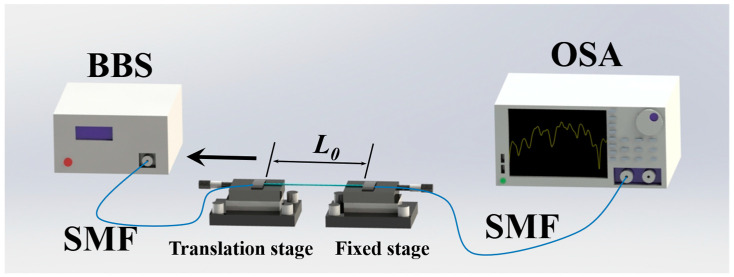
Schematic diagram of strain experimental device.

We also measured the strain characteristics of the proposed sensor. Figure 9 shows the setup diagram of the strain experiment. The sensor is placed on the fiber holders of the two stages, and the strain is achieved by adjusting the left translation stage. Two ends of the sensor are connected to BBS and OSA, respectively, to monitor the changes in the spectrum in real time. The axial strain can be calculated by the following formula:

(6) $$\Delta \varepsilon =\frac{\Delta L}{L_{0}},$$

where L_0_ is the distance between the two initial stages and ΔL is the shifted distance of the translation stage.

**Figure 10:**
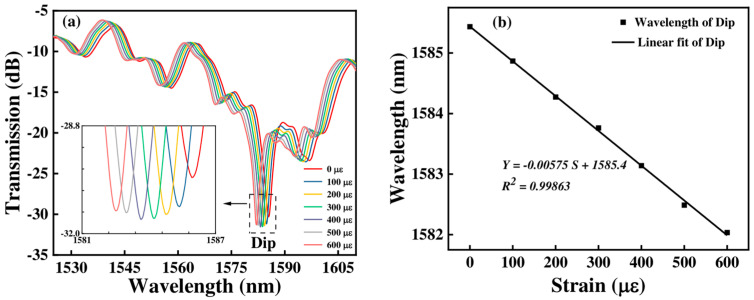
(**a**) The transmission spectra of Sensor 1 at various strains; (**b**) relationship between wavelength and strain.

Figure 10 shows the transmission spectrum and the corresponding linear fitting curve of Sensor 1 with the strain changing. We can see that when the strain changes from 0 to 600 με, the wavelength dip has a blue shift from 1585.435 nm to 1582.035 nm, and its intensity also decreases. Figure 10b shows the strain sensitivities of Sensor 1 in terms of wavelength. We can obtain that the strain sensitivity of Sensor 1 is −5.75 pm/με.

**Figure 11:**
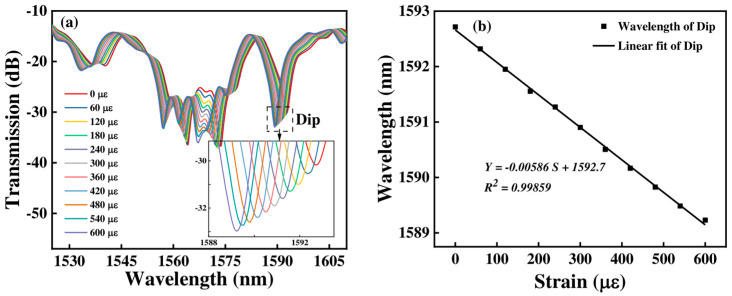
(**a**) The transmission spectra of Sensor 2 at various strains; (**b**) linear fitting response between wavelength and strain.

For Sensor 2, when its strain changes from 0 to 600 με, the transmission spectrum is shown in Figure 11a. The transmission spectra show a blue shift from 1592.717 nm to 1589.232 nm for the Dip, and the total wavelength offset is −3.485 nm. From Figure 11b, we can obtain that the strain sensitivity is −5.86 pm/με with a linearity of 0.99859 for the dip.

**Figure 12:**
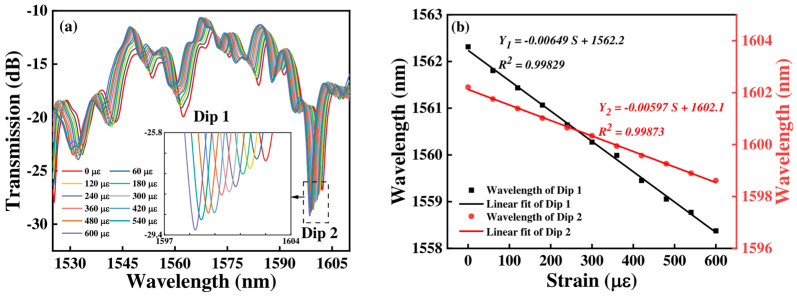
(**a**) The transmission spectra of Sensor 3 at various strains; (**b**) linear fitting responses between wavelength and strain.

Figure 12 shows the transmission spectrum and the corresponding linear fitting curve of Sensor 3 with the strain variation. When the strain increases, the transmission spectra of Sensor 3 are blue shifted. The wavelength shift from 1562.315 nm to 1558.377 nm, and 1602.208 nm to 1598.610 nm for Dip 1 and Dip 2, and the wavelength offsets are −3.938 nm and −3.598 nm, respectively. From Figure 12b, we can obtain that the strain sensitivities are −6.49 pm/με, and −5.97 pm/με with a linearity of 0.99829, and 0.99873 for Dip 1, and Dip 2, respectively.

By comparing the results in Figure 10, Figure 11 and Figure 12, we can see that with the FCF length increasing, the strain sensitivity of the sensor gradually increases, and the maximum strain sensitivity can achieve −6.49 pm/με. This is because lengthening the FCF increases the interference length of the multi-core fiber, leading to a small stress change that affects the interference fringe and sensor sensitivity.

By comparing the curvature and strain sensing, we found that these phenomena can be detected by different characteristics of the proposed sensor. During curvature changes, the wavelength remains nearly constant. So, if only intensity changes without any variation in wavelength, it can be considered the curvature sensing under constant strain. If there is a shift in wavelength, we can detect the wavelength variation to determine strain fluctuations. Therefore, the cross-sensitivity of curvature and strain can be solved by identifying the loss of intensity and spectral characteristics. For the potential impact of the external environment, we can use high-quality and stable packaging materials to protect the sensor from the direct impact of external environmental changes.

**Figure 13:**
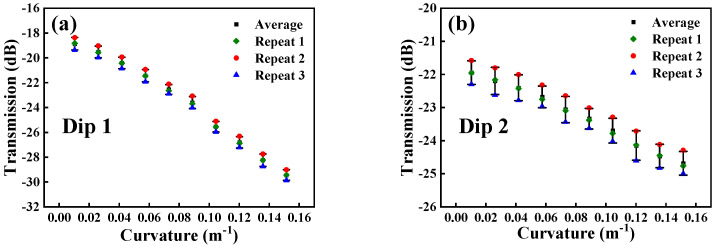
Curvature repeatability experiments. (**a**) Dip 1; (**b**) Dip 2.

**Figure 14:**
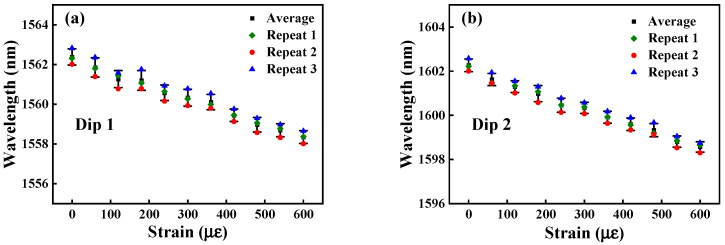
Strain repeatability experiments. (**a**) Dip 1; (**b**) Dip 2.

In order to confirm the reliability of the sensor, we conducted three repetitive experiments on Sensor 3 with the highest sensitivity. As shown in Figure 13 and Figure 14, we found that there was no significant change in the experimental results, which provides a reliable basis for its practical application.

To improve the performance of the proposed sensor, higher-precision splicing and fusion technology can be employed to increase the splice and fusion accuracy, thereby improving the light coupling and reducing the loss. Additionally, corrosion-resistant and highly stable packaging materials can be used to increase the stability of the sensor. Furthermore, machine learning methods can be leveraged to extend the sensor’s dynamic measurement range [20].

To better reflect the sensitivity of this MZI structure, we compared the sensitivity of existing MZI sensors. As can be seen from Table 1, our sensor has high sensitivity in measuring curvature and strain and has good prospects in practical applications.

## 4. Conclusions

In summary, we proposed a highly sensitive MZI sensor consisting of NCF and FCF, where the proposed sensor has a high response to curvature and strain. By comparing sensors with three different NCF lengths, the results show that 7.5 mm NCF can better couple light into the cores of FCF and generate a better interference fringe. We conducted experimental studies on the three groups of sensors, and the experimental results showed that the maximum curvature and strain sensitivities of −78.04 dB/m^−1^ and −6.49 pm/με were achieved when the curvature range varies in the range of 0.0104 m^−1^–0.1515 m^−1^ and strain range of 0–600 με with good linearity, respectively. Due to its high sensitivity and small size, the proposed sensor has good application potential in curvature and strain measurement, with simple detection and low cost.

## Figures and Tables

**Table 1:** Comparison of the sensing characteristics with previously reported sensors.

Structure	Sensitivity of Curvature	Sensitivity of Strain	Reference
SMF-FCF-SMF	18.75 nm/m^−1^	--	[9]
SMF-NCF-SCF-NCF-SMF	10.22 dB/m^−1^	--	[13]
SMF-FCF-SMF	20.18 nm/m^−1^	1.78 pm/με	[21]
FBG-SMF-FCF-SMF	15.57 pm/m^−1^	--	[22]
SMF-MMF-HCF-MMF-SMF	19.58 dB/m^−1^	--	[23]
SMF-NCF-FCF-NCF-SMF	−78.04 dB/m^−1^	−6.49 pm/με	This Work

## Data Availability

The data presented in this study are available from the corresponding author upon reasonable request.
